# Development and Validation of a Serum Metabolomic Signature for Endometrial Cancer Screening in Postmenopausal Women

**DOI:** 10.1001/jamanetworkopen.2020.18327

**Published:** 2020-09-28

**Authors:** Jacopo Troisi, Antonio Raffone, Antonio Travaglino, Gaetano Belli, Carmen Belli, Santosh Anand, Luigi Giugliano, Pierpaolo Cavallo, Giovanni Scala, Steven Symes, Sean Richards, David Adair, Alessio Fasano, Vincenzo Bottigliero, Maurizio Guida

**Affiliations:** 1 Department of Medicine, Surgery and Dentistry, Scuola Medica Salernitana, University of Salerno, Baronissi, Salerno, Italy; 2 Theoreo, Montecorvino Pugliano, Salerno, Italy; 3 European Biomedical Research Institute of Salerno, Salerno, Italy; 4 Department of Neurosciences and Reproductive and Dentistry Sciences, University of Naples Federico II, Naples, Italy; 5 Department of Advanced Biomedical Sciences, University of Naples Federico II, Naples, Italy; 6 Lega Italiana per la Lotta contro i Tumori, Avellino Section, Avellino, Italy; 7 Università degli Studi di Milano–Bicocca, Milano, Italy; 8 Faculty of Medicine, University of Geneva Medical School, Geneva, Switzerland; 9 Department of Physics, University of Salerno, Fisciano, Salerno, Italy; 10 Istituto Sistemi Complessi–Consiglio Nazionale delle Ricerche, Rome, Italy; 11 Hosmotic, Vico Equense, Naples, Italy; 12 Department of Chemistry and Physics, The University of Tennessee at Chattanooga; 13 Department of Obstetrics and Gynecology, College of Medicine, University of Tennessee College of Medicine at Chattanooga; 14 Department of Biology, Geology and Environmental Sciences, The University of Tennessee at Chattanooga

## Abstract

**Importance:**

Endometrial carcinoma (EC) is the most commonly diagnosed gynecologic cancer. Its early detection is advisable because 20% of women have advanced disease at the time of diagnosis.

**Objective:**

To clinically validate a metabolomics-based classification algorithm as a screening test for EC.

**Design, Setting, and Participants:**

This diagnostic study enrolled 2 cohorts. A multicenter prospective cohort, with 50 cases (postmenopausal women with EC; International Federation of Gynecology and Obstetrics stage I-III and grade G1-G3) and 70 controls (no EC but matched on age, years from menopause, tobacco use, and comorbidities), was used to train multiple classification models. The accuracy of each trained model was then used as a statistical weight to produce an ensemble machine learning algorithm for testing, which was validated with a subsequent prospective cohort of 1430 postmenopausal women. The study was conducted at the San Giovanni di Dio e Ruggi d’Aragona University Hospital of Salerno (Italy) and Lega Italiana per la Lotta contro i Tumori clinic in Avellino (Italy). Data collection was conducted from January 2018 to February 2019, and analysis was conducted from January to March 2019.

**Main Outcomes and Measures:**

The presence or absence of EC based on evaluation of the blood metabolome. Metabolites were extracted from dried blood samples from all participants and analyzed by gas chromatography–mass spectrometry. A confusion matrix was used to summarize test results. Performance indices included sensitivity, specificity, positive and negative predictive values, positive and negative likelihood ratios, and accuracy. Confirmation or exclusion of EC in women with a positive test result was by means of hysteroscopy. Participants with negative results were followed up 1 year after enrollment to investigate the appearance of EC signs.

**Results:**

The study population consisted of 1550 postmenopausal women. The mean (SD) age was 68.2 (11.7) years for participants with no EC in the training cohort, 69.4 (13.8) years for women with EC in the training cohort, and 59.7 (7.7) years for women in the validation cohort. Application of the ensemble machine learning to the validation cohort resulted in 16 true-positives, 2 false-positives, and 0 false-negatives, and it correctly classified more than 99% of samples. Disease prevalence was 1.12% (16 of 1430).

**Conclusions and Relevance:**

In this study, dried blood metabolomic profile was used to assess the presence or absence of EC in postmenopausal women not receiving hormonal therapy with greater than 99% accuracy.

## Key Points

**Question:**

Is combining the blood metabolomic signature of endometrial carcinoma with an ensemble machine learning algorithm a useful system for building a screening test for endometrial cancer?

**Findings:**

In this diagnostic study that included 1550 postmenopausal women, the proposed screening test correctly identified all 16 women with endometrial cancer, with 2 false-positive results and 0 false-negative results.

**Meaning:**

The results of this study suggest that the metabolomic profile of a blood sample could provide a noninvasive and accurate screening test with high sensitivity and specificity for endometrial cancer.

## Teaser

This diagnostic test validates a metabolomics-based classification algorithm as a screening test for endometrial carcinoma.

## Introduction

Endometrial carcinoma (EC) is the most common malignant tumor of the female genital tract in high-income countries and the sixth most frequent in women worldwide, with an estimated 380 000 new cases globally in 2018.^1^ Incidence is increasing, owing primarily to the increasing prevalence of risk factors, such as obesity, diabetes, metabolic syndrome, and others.^2,3,4^ Sheikh et al^5^ estimated an incidence rate of 42 cases per 100 000 individuals in 2030, with a 55% increase compared with 2010.

Five-year survival rates of patients with EC are inversely associated with their International Federation of Gynecology and Obstetrics (FIGO) stage at diagnosis: from 85% at stage I to 25% at stage IV.^6^ Thus, lower risk of mortality and longer disease-free survival rates appear to be associated with earlier diagnosis, highlighting the crucial role of screening programs.^7^ Presently, there is not a clinically validated test for EC screening.

Since the pioneering studies of Dr Papanicolaou regarding screening tests based on cervical cytology for the screening of cervical carcinoma,^8^ attempts have been made to develop cytological studies using cervical liquids for EC screening. The limiting factor for using the endocervical smear for EC detection is the high frequency of false-negative results. It is difficult to microscopically evaluate cervical fluids because of the low abundance of endometrial malignant cells that can be detected, even in patients with advanced EC.^9^ Moreover, the acidic vaginal environment modifies the exfoliated endometrial cells to the extent that positive identification of tumor cells becomes difficult.

Transvaginal ultrasound screening using endometrial thickness (ET) was also proposed for symptomatic postmenopausal women not receiving hormonal treatment. Unfortunately, an ET cutoff of 5 mm showed lower sensitivity (77.1%; 95% CI, 67.8%-84.3%) and specificity (85.8%; 95% CI, 85.7%-85.9%).^10^

Therefore, new screening tests combining cytological and genetic evaluation have been proposed, with interesting preliminary results.^11,12^ However, a validation of these systems on a large population is still lacking, and no screening test for EC is feasible to date. Moreover, genetic evaluation is costly and time-consuming and needs histological correlates.

In a previous study,^13^ we found a serum metabolomic signature able to discriminate patients with EC from healthy women and from women with other endometrial diseases. Metabolomics is inexpensive, fast, and noninvasive—all characteristics of an ideal screening test.^14^ However, further validation using a larger sample set is necessary to establish metabolomics as a viable screening test. Thus, the aim of this study was to test the effectiveness of the already reported metabolomics signature as an EC screening test using blood samples from a large (ie, >1400) postmenopausal, general population.

## Methods

### Study Protocol

The diagnostic study was designed as a multicenter prospective study following an a priori study protocol. The study was reported according to the Standards for Reporting of Diagnostic Accuracy (STARD) reporting guideline.

Two separate clinical enrollments were used for this study to obtain blood samples for metabolomic analyses. The first was a training cohort that collected blood samples from 50 women with an established diagnosis of EC with any FIGO stage (I-III), histological grade, and histotype (before surgery and/or other treatments). These participants were matched on age, years from menopause, tobacco use, and comorbidities with 70 controls. These samples were used to train the classification models in the same manner as our previous reports.^13,15,16^ The diagnostic performances of the resulting models were evaluated using cross-validation. The second cohort collected samples from a population with unknown EC status to test the screening performance of the trained models (ie, independent test set). Enrollment in the training set was completed at the San Giovanni di Dio e Ruggi d’Aragona University Hospital of Salerno (Italy), while the second enrollment was conducted at the Lega Italiana per la Lotta contro i Tumori clinic in Avellino (Italy).

Sample collection strictly adhered to the guidelines outlined in the Declaration of Helsinki.^17^ The study received approval by the institutional review board Comitato Etico Campania Sud. All participants provided written informed consent for the use of their biospecimens for research purposes, and all data were anonymized to prevent the identification of the subjects.

Inclusion criteria were as follows: women aged between 50 and 80 years with postmenopausal status. Women with a previous diagnosis of cancer (in any organ), who had previously undergone hysterectomy, who were receiving hormonal therapy for menopausal symptoms, or who were receiving immunosuppressive therapy before enrollment were excluded.

After metabolomic assessment of blood samples from the independent test set, women with a positive metabolomic screening test (ie, classified by the ensemble machine learning [EML] model as being positive for EC) underwent a histological examination of endometrial biopsy to either confirm or exclude the EC suspicion. The remaining participants underwent a phone questionnaire 12 months after enrollment to investigate the appearance of EC signs (eg, abnormal uterine bleeding) during this period. Women who endorsed EC signs underwent ultrasonography scan and hysteroscopy or dilation and curettage to obtain endometrial specimens for histological examination.

### Demographic Characteristics and Clinical Assessment

For each included woman, data regarding age, ethnicity, weight, height, waist circumference, tobacco and alcohol use, heart rate, blood pressure, present or past occupation, pharmaceutical treatments, and abnormal uterine bleeding were collected. Moreover, gynecological anamnesis was assessed with particular attention to uterine, ovarian, and vaginal diseases. For patients with EC in the training cohort, EC histotype, FIGO grade and stage, and oncological history were also recorded.

Exposure to cigarette smoke was assessed with methods using the most recent findings in the literature.^18^ For example, the pack-year (or tabagic index) parameter estimates the load of tobacco that the participant used during her life as a first-hand smoker. The formula used for the calculation is the mean number of cigarettes per day times the number of years of active smoking divided by 20 (ie, the number of cigarettes in a pack). According to the National Comprehensive Cancer Network, smoke-related risk is stratified in the 4 following classes^19^: low risk, younger than 50 years of age and fewer than 20 pack-years; moderate risk, aged 50 years or older and 20 or more pack-years of active or secondary smoking in the absence of other risk factors; high risk, aged 50 years or older and 20 or more pack-years of active smoking in the presence of another risk factor except passive smoking; very high risk, aged 55 years or older and 30 or more pack-years of active smoking (unless the individuals has quit smoking for more than 15 years). Consistent with this classification, we estimated the number of participants at very high risk compared with both the totality of all recruited participants and the participants with current tobacco use.

### Blood Collection

Metabolomic investigations were performed by using dried blood spot (DBS) samples. Five blood drops, taken by means of digital puncture, were collected using the Whatman Protein Saver 903 Card (GE Healthcare). This method is the least invasive sampling system to obtain blood samples to date.

### Untargeted Metabolomics Analysis

Metabolomics analysis was performed according to Troisi et al,^13^ with minor modifications. Metabolomes were extracted from DBS using the MetaboPrep GC Kit (Theoreo) within 5 days of collection. Two separate extraction steps were used; the first consisted of vortex-mixing at 1250 rpm of a 5 mm diameter DBS with the extraction solution for 30 minutes. The second consisted of ultrasonograph-assisted solubilization of the same mixture (DBS paper and extraction solution) for 30 minutes at 30 °C. The extracted samples were then derivatized and analyzed with gas chromatography–mass spectrometry.^13^

### Statistical Analysis

#### Demographic and Clinical Data

Normal distribution of data was verified using the Kolmogorov-Smirnov test. Normally distributed data were reported as means and SDs, and the *t* test was used for univariate comparisons. Study data were collected and managed using REDCap electronic data capture tools,^20^ hosted at the Istituto Nazionale di Fisica Nucleare in the University of Salerno. Statistical analysis was performed using R version 3.5.2 (R Project for Statistical Computing). A 2-tailed *P* <.05 was considered statistically significant.

#### Sample Size Calculation

The target sample size for each enrollment was calculated following different strategies. Details of the calculations are reported in the eAppendix in the Supplement. Based on the sample size calculation, we had to enroll at least 50 participants for each group (EC and no EC) in the training set and at least 1350 participants in the second enrollment.

#### Classification Model Building

An EML model, based on a voting scheme statistically weighted by the individual classification accuracy of 10 different classification models (ie, decision tree, partial least–square discriminant analysis, naive Bayes, random forest, k–nearest neighbor, artificial neuronal network, support vector machines, linear discriminant analysis, logistic regression, and deep learning), was built according to Troisi et al,^13^ using R version 3.5.2 and RapidMiner Studio version 8.1 (RapidMiner). Samples collected in the training set were used to train the 10 individual models, and those results were used to generate the EML model. Application of the EML model using the metabolomic profile of blood samples from the 1430 participants with unknown EC status was the screening test referred to in this article.

#### EC-EML Score

For each classification model, cross-validation accuracy was evaluated. For each sample and for each classification model, the classification confidence was also evaluated. From these parameters, a model score was calculated by multiplying the classification accuracy by the classification confidence. Scores of participants classified as having EC were considered as is, while scores of control participants were multiplied by −1. Finally, an EC-EML score was calculated for each sample by summing all individual classification model scores.

The areas under receiver operating characteristic (ROC) curves were calculated to evaluate the ability of the EC-EML score to predict the presence of EC. A cutoff point was evaluated as the score maximizing the Youden Index (sensitivity + specificity − 1). We used the nonparametric approach of DeLong et al^21^ to compare the areas under the ROC curves. Moreover, an EC-EML score of 0 was considered a cutoff value to account for situations in which the votes for and against EC were equal.

#### Screening Test Performance Evaluation

The overall diagnostic performance of the proposed EML-based screening test was investigated using a confusion matrix to summarize the results using the metabolomic profiles of samples obtained by means of the second enrollment. Performance metrics derived from the matrix included sensitivity, specificity, positive and negative predictive values, positive and negative likelihood ratios, and accuracy. The formulas used for these parameters are reported in eTable 1 in the Supplement.

## Results

### Participant Characteristics

According to the sample size calculation, 120 women were included in the training set: 50 (41.7%) with EC (mean [SD] age, 69.4 [13.8] years) and 70 (58.3%) with no EC (mean [SD], 68.2 [11.7] years). Of the 50 women with EC, 27 (54%) had stage I disease, 16 (32%) had stage II disease, and 7 (14%) had stage III disease, according to the FIGO classification. Regarding tumor grading, 11 (22%) were G1; 29 (58%), G2; and 10 (20%), G3.

**Figure 1.**
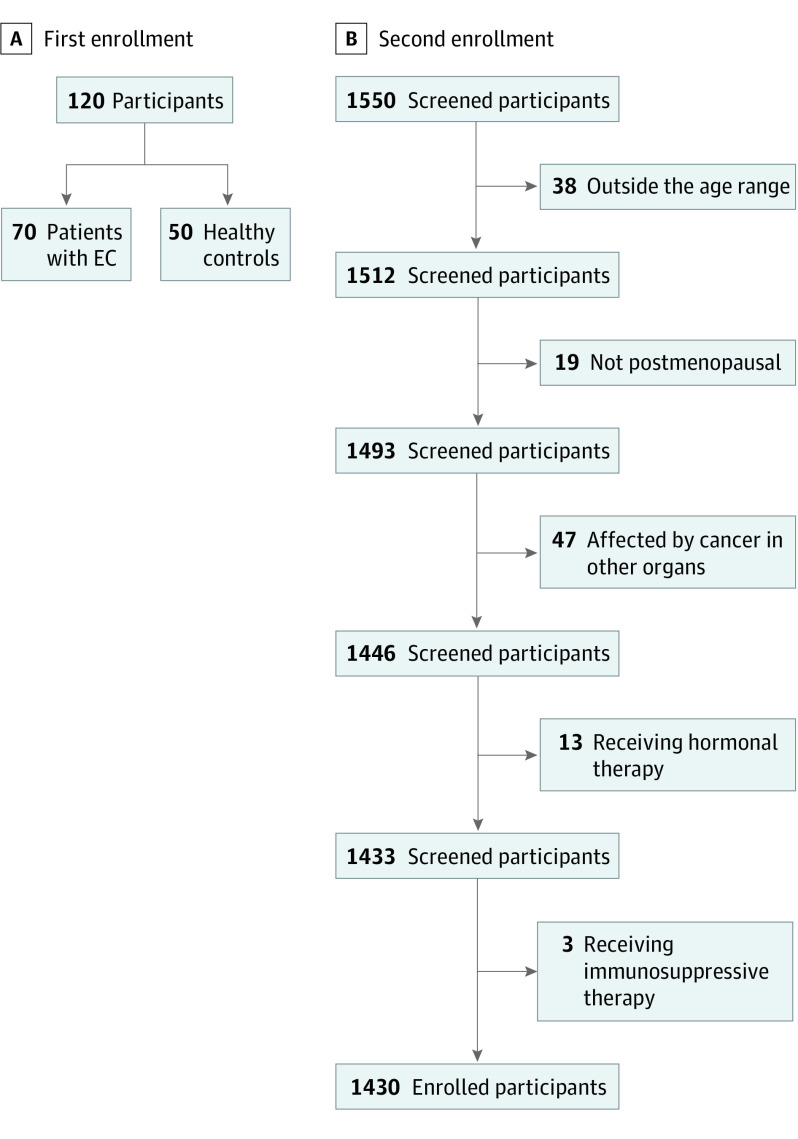
Enrollment Flow Chart The first enrollment was the training set, and the second was the test set. EC indicates endometrial cancer.

The second enrollment (the test set) consisted in the collection of samples from 1430 women (mean [SD] age, 59.7 [7.7] years) with unknown EC status from the general population. The enrollment flow charts and participant characteristics are reported in Figure 1 and Table 1, respectively.

**Table 1.** Study Population Features

Characteristic	Participants, No. (%)		
	Training set		Test set (n = 1430)
	With no EC (n = 70)	With EC (n = 50)	
Age, mean (SD), y	68.2 (11.7)	69.4 (13.8)	59.7 (7.7)^a^
Age at last period, mean (SD), y	52.3 (3.9)	50.1 (4.4)^a^	49.8 (4.4)^a^
Time from menopause, mean (SD), y	11.5 (7.4)	11.9 (8.8)	12.0 (8.2)
Blood pressure, mean (SD), mm Hg			
Systolic	128.5 (4.1)	129.1 (6.5)	120.5 (11.2)^a^
Diastolic	81.2 (5.3)	82.9 (6.0)	75.6 (7.8)^a^
Hypertension			
Yes	27 (38.6)	23 (45.0)	582 (40.7)
No	43 (61.4)	28 (55.0)	848 (59.3)
Heart rate, mean (SD), bpm	78.5 (4.3)	81.0 (6.7)^b^	70.4 (6.4)^a^
Weight, mean (SD), kg	73.2 (10.4)	75.6 (11.8)	69.1 (12.5)^b^
Height, mean (SD), cm	162.1 (4.8)	160.7 (5.1)	160.6 (5.7)
BMI			
Mean (SD)	27.6 (4.3)	29.3 (4.9)^c^	26.8 (4.6)
Underweight, No. (%)	1 (1.4)	1 (2.0)	6 (0.4)
Normal weight, No. (%)	13 (18.6)	9 (18.0)	285 (19.9)
Overweight, No. (%)	41 (58.6)	29 (57.5)	829 (58.0)
Obesity, No. (%)	15 (20.8)	12 (23.0)	310 (21.7)
Endometrial thickness, mean (SD), mm	<4	22.5 (14.0)^a^	<4
Abdominal circumference, mean (SD), cm	78.2 (16.5)	82.3 (18.9)	79.5 (24.5)
Tobacco use			
Current	15 (21.4)	13 (26.0)	349 (24.4)
Never	40 (57.1)	26 (52.0)	831 (58.1)
Former	15 (21.5)	11 (22.0)	250 (17.5)
≥30 Packages/y among all participants	4 (5.3)	2 (4.0)	83 (5.8)
≥30 Packages/y among participants with current tobacco use, No./total No. (%)	4/15 (26.7)	2/13 (15.4)	83/349 (23.8)
Cigarette packs per y, mean (SD)	12.2 (7.6)	15.7 (12.8)	16.4 (14.1)
Metrorrhagia in last year			
Yes	2 (2.8)	47 (94.0)^a^	51 (3.6)
No	68 (97.2)	3 (6.0)^a^	1379 (96.4)
Diabetes			
Yes	6 (8.6)	5 (10.0)	127 (8.9)
No	64 (91.4)	45 (90.0)	1303 (91.1)
Hypertriglyceridemia			
Yes	4 (5.7)	4 (8.0)	76 (5.3)
No	66 (94.3)	46 (92.0)	1354 (94.7)
Hyperuricemia			
Yes	1 (1.4)	1 (2.0)	13 (0.9)
No	69 (98.6)	49 (98.0)	1417 (99.1)
Vasculopathies			
Yes	6 (8.6)	5 (10.0)	142 (9.9)
No	64 (91.4)	45 (90.0)	1288 (90.1)
Cholecystectomies			
Yes	6 (8.6)	6 (12.0)	134 (9.4)
No	64 (91.4)	44 (88.0)	1296 (90.6)
CABG			
Yes	0 (0.0)	1 (2.0)	11 (0.8)
No	70 (100.0)	49 (98.0)	1419 (99.2)

Abbreviations: BMI, body mass index (calculated as weight in kilograms divided by height in meters squared); CABG, coronary artery bypass graft surgery; EC, endometrial carcinoma.

^a^ *P* < .001.

^b^ *P* < .01.

^c^ *P* < .05.

Participants from the second recruitment were younger than the women in the control group (mean [SD] age, 59.7 [7.7] years vs 68.2 [11.7] years; *P* < .001) and presented lower values of both systolic and diastolic blood pressure (mean [SD] systolic blood pressure: 120.5 [11.2] mm Hg vs 128.5 [4.1] mm Hg; *P* < .001; mean [SD] diastolic blood pressure: 75.6 [7.8] mm Hg vs 81.2 [5.3] mm Hg; *P* < .001) and heart rate (mean [SD] heart rate, 70.4 [6.4] bpm vs 78.5 [4.3] bpm; *P* < .001). On the contrary, women with EC had higher body mass index (calculated as weight in kilograms divided by height in meters squared) and more common abnormal uterine bleeding compared with the control group (mean [SD] body mass index: 29.3 [4.9] vs 27.6 [4.3]; *P* = .046; participants with metrorrhagia in last year: 47 [94.0%] vs 2 [2.8%]; *P* < .001). No significant differences in tobacco use were found among all enrolled participants.

### Metabolomic Analyses

**Figure 2.**
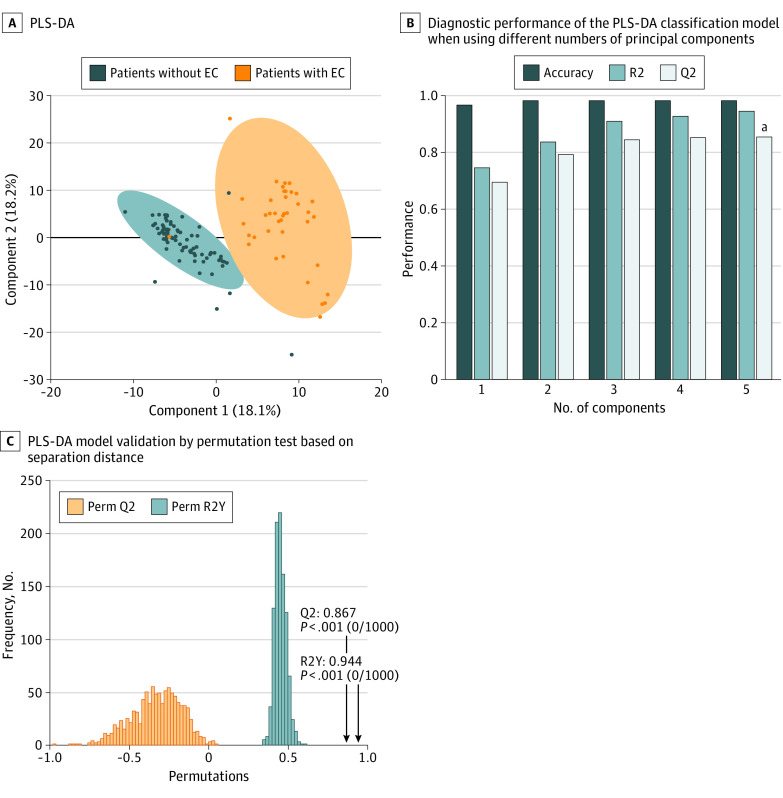
Partial Least Square–Discriminant Analysis (PLS-DA) Model A, The figure illustrates separation between the training set data, indicating that the measured metabolomic profiles of patients with vs without endometrial carcinoma (EC). B, The best performance (denoted with *a*) was achieved when using 5 principal components as the basis for classification. C, PLS-DA model validation by permutation test based on separation distance. Perm indicates permutation-based.

Gas chromatography–mass spectrometry analysis of 1550 derivatized samples was able to detect 293 peaks in each specimen; some of these peaks were not further considered because they were not consistently found in at least 75% of samples, were too low in concentration, or were of too poor spectral quality to be confirmed as metabolites. As a result, a total of 268 endogenous metabolites were consistently detected. A comparison of the metabolomes between the training set and the test set is shown in the eFigure in the Supplement. Figure 2 reports the partial least square–discriminant analysis scatterplot representation of this classification model based on the training set; very little overlap between cases and controls is observed. The permutation test statistically assessed the class separation of metabolomes by visually illustrating and measuring the statistical significance of case and control metabolome separations and lack of overfittings among patients in the training set with vs without EC. Performance indices based on 1000 permutations were *R*^2^_Y_ = 0.944 and *Q*^2^_Y_ = 0.867.

Table 2 reports the diagnostic performance for each classification model when applied to the training set and test set. Performance metrics were evaluated using the technique of cross-validation. The best accuracies for training set were obtained from k–nearest neighbor and logistic regression models (100%), while linear discriminant analysis showed the lowest score (83.3%). In addition, the EML model showed no classification errors (100%). Samples from the second enrollment were classified according to the following ensemble rules: each sample was first classified as EC positive or EC negative by each classification model. The classification cross-validation accuracy of each model was then used as a statistical weighting for each result. These results were then ensembled by assigning the overall class based on membership probability of greater than 50%.

**Table 2.** Classification Models and EML Diagnostic Performance

Enrollment	Classification model	% (SE)				Likelihood ratio		Accuracy
		Sensitivity	Specificity	PPV	NPV	Positive	Negative	
Training set	Decision tree	95.0 (3.4)	97.5 (1.7)	95.0 (3.4)	0.97.5 (1.7)	38.0	0.05	96.7
	Naive Bayes	65.0 (7.5)	96.3 (2.1)	89.7 (5.7)	84.6 (3.8)	17.3	0.36	85.8
	Random forest	87.5 (5.2)	100.0 (0.0)	100.0 (0.0)	94.1 (2.6)	ND	0.13	95.8
	k–Nearest neighbors	100.0 (0.0)	100.0 (0.0)	100.0 (0.0)	100.0 (0.0)	ND	0.00	100.0
	Artificial neural network	92.5 (4.2)	100.0 (0.0)	100.0 (0.0)	96.4 (2.0)	ND	0.08	97.5
	Linear discriminant analysis	50.0 (7.9)	100.0 (0.0)	100.0 (0.0)	80.0 (4.0)	ND	0.50	83.3
	Support vector machine	55.0 (7.9)	100.0 (0.0)	100.0 (0.0)	81.6 (3.9)	ND	0.45	85.0
	Linear regression	100.0 (0.0)	100.0 (0.0)	100.0 (0.0)	100.0 (0.0)	ND	0.00	100.0
	Deep learning	97.5 (2.5)	98.8 (1.2)	97.5 (2.5)	98.8 (1.2)	78.0	0.03	98.3
	Partial least squares–discriminant analysis	92.5 (4.2)	100.0 (0.0)	100.0 (0.0)	96.4 (2.0)	ND	0.08	97.5
	EML	100.0 (0.0)	100.0 (0.0)	100.0 (0.0)	100.0 (0.0)	ND	0.00	100.0
Test set	EML	100.0 (0.0)	99.9 (1.0)	88.9 (7.4)	100.0 (0.0)	707.0	0.0	99.9

Abbreviations: EML, ensemble machine learning; ND, not determinable; NPV, negative predictive value; PPV, positive predictive value.

**Figure 3.**
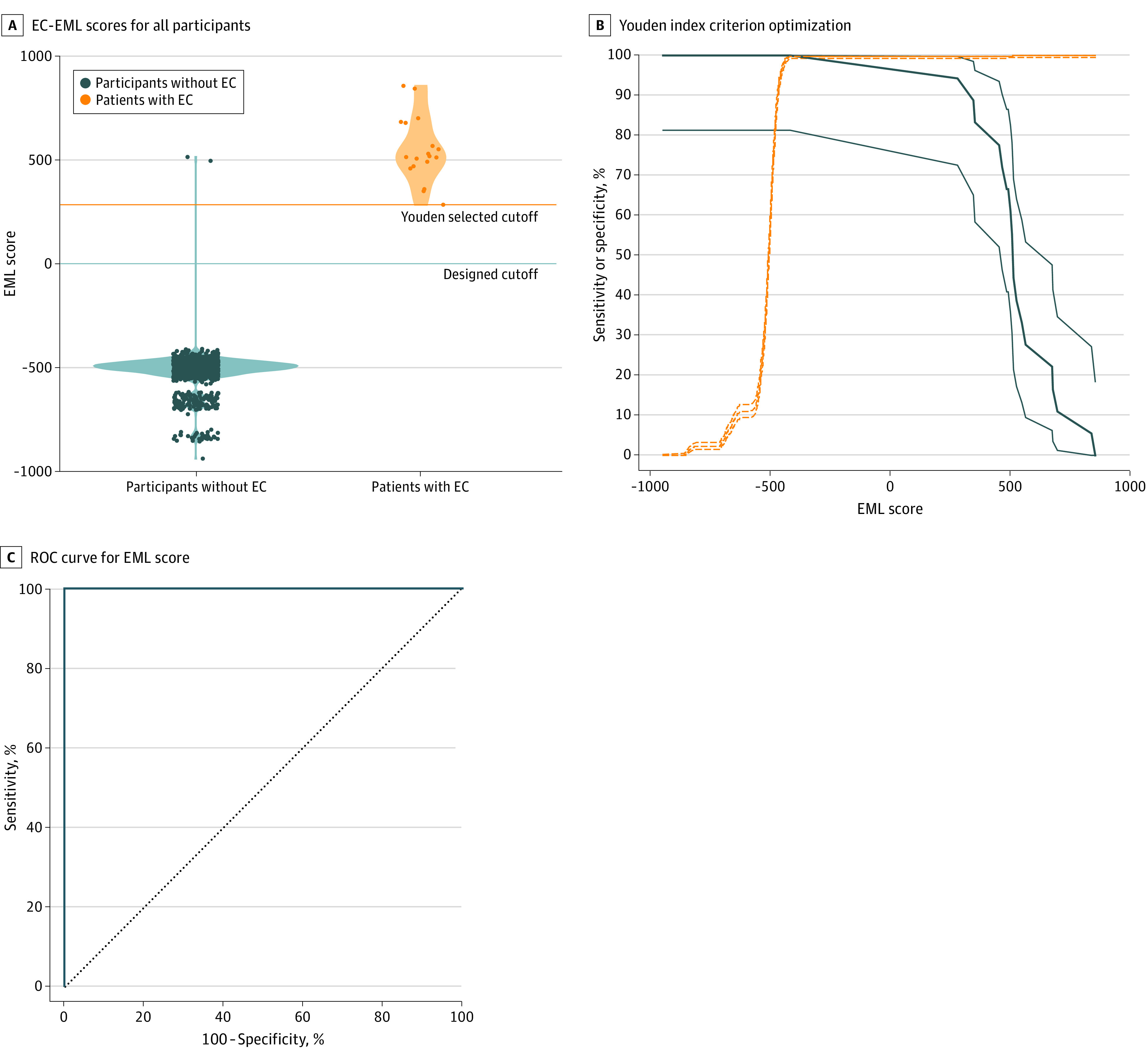
Endometrial Cancer Ensemble Machine Learning (EC-EML) Score A, Circles represent EC-EML scores. The orange line represents the cutoff value evaluated by Youden index optimization, while the blue line represents EC-EML score of 0, which was the projected cutoff. B, The dotted lines represent the sensitivity while the continuous lines represent the specificity. C, Receiver operating characteristic (ROC) curve of the EC-EML score.

The results are fully presented in eTable 2 in the Supplement and synthetized in Table 2. Overall, 18 participants were classified by the EML as having EC. Of these, 16 (88.9%) were confirmed to have EC through histological examination of endometrial samples, while 2 (11.1%) were false-positive results (Figure 3). Disease prevalence of 1.12% (16 of 1430). Of the 16 women with EC, 12 (75.0%) had stage 1A (cancer found only in the endometrium or <50% of the myometrium), 3 (18.8%) had stage 1B (cancer spread to ≥50% of the myometrium without involvement of other organs), while only 1 (6.2%) had stage 2 (cancer involving the cervical stroma but not to other parts of the body). No participants with EML negative results reported signs or symptoms of EC presence at the phone questionnaire 12 months after the enrollment. According to the voting scheme of the EML, the cutoff value can be evaluated as an EC-EML score of 0, which is the balance value between having EC (EC-EML score >0) or being in the control group (EC-EML score <0) classification. Cutoff value was also evaluated by the Youden index resulting, in an EC-EML score of greater than 284.87. The overall performance for this metabolomics-based EC screening test for both cutoff values resulted in a mean (SD) accuracy of 99.86% (0.89%).

## Discussion

This study aimed to validate a previously reported metabolomics signature as a screening test for EC on a large study population. The screening test was based on an EML algorithm that sums the voting results of 10 different classification models, statistically weighted by each models’ classification accuracy. The overall screening test showed no false-negative results and 2 false-positive results based on 1430 analyzed samples. The accuracy was 99.86%.

Despite great efforts in EC screening research, no screening tools have been validated to date. Histological examination of endometrial specimens by hysteroscopy or dilation and curettage remains the criterion standard in the diagnosis of EC. However, such a diagnosis is limited by sampling invasiveness, sampling error (particularly for early-stage disease), high cost, procedure-related complications, and poor reproducibility of the histological examination even when performed by expert pathologists.^22,23^ Furthermore, some women consider this procedure embarrassing, another factor for a late-stage diagnosis.^23,24^ The high survival rate when diagnosed at an early stage highlights the importance of a noninvasive screening system.^1,25^

Metabolomics is a noninvasive, inexpensive, and high-throughput technology that can measure hundreds of different metabolites simultaneously in a small volume of fluid or tissue.^26^ Metabolomics can be based on mass spectrometry that, both as a standalone and combined with chromatographic systems, is widely used in analytical chemistry and clinical laboratories for toxic effect studies as well as in the diagnosis of inherited metabolic disorders. To date, several applications of this approach in early diagnosis of human disease have been reported,^15,16,27,28,29^ with particular regard to oncological diseases.^13,14,30,31^ Among the most important limitations in clinical application of the already reported metabolomics signatures is the lack of diagnostic validation on large patient cohorts.

We report the results obtained in a large population that could be interpreted as a clinical validation of the screening test under real conditions. We found a very high accuracy, which exceeds that expected for a screening test. Such results seem to be better than those reported for the most promising EC screening tools to date.^11,12^ In fact, a sensitivity of 100% with a mean (SD) specificity of 99.86% (0.10%) has not been previously reported. Furthermore, the costs required for this screening test would be lower than other promising tests, which are based on expensive genomic analyses. Finally, compared with previously reported EC screening tests, this test is minimally invasive because it not only avoids uterine tissue sampling but is also based on the least invasive method to obtain blood samples to date. Further studies may be necessary to confirm these findings on even larger cohorts. Regardless, our results indicate the proposed approach results in early-stage cancer detection, urging a broad adoption of such protocol for routine screening.

Recruitment for the test set was conducted in a cancer clinic while the women with EC and their matched controls were enrolled in a hospital before any surgery or drug therapy. The enrolled populations were slightly different in terms of anthropometric and clinical features. Some of these differences may reflect the different enrolling environments. For example, the participants with unknown EC status showed lower blood pressure and heart rate, and they were enrolled in a cancer clinic. This could reflect lower stress and less discomfort related to the more comfortable and relaxed environment of the clinic. Importantly, these small differences did not affect the metabolomes, as shown by the fact that a principal component analysis did not show differences in the metabolomes of the 2 enrolled populations (eFigure in the Supplement).

To our knowledge, this may be the first study that enrolled a large population (n = 1430) to test a metabolomic approach as a screening system for EC. In addition, our validation cohort consisted of women from a nonprofit oncological clinic, which is an ideal study population to assess screening tests. Indeed, these women did not present any EC signs or symptoms but had several EC-related risk factors. This study design also allowed us to assess the overall predictive ability of the test by calculating the areas under ROC curves. Other strengths of our study include detailed and standardized metabolomics analysis, and implementation of a minimally invasive blood sampling procedure. Moreover, the reported EML algorithm showed good performance in EC detection.

### Limitations

This study has limitations. First, there is no criterion standard to evaluate participants with no EC (classified as control by the EML), who only underwent a phone questionnaire to determine the presence of any EC symptoms 12 months after enrollment. In fact, a definitive diagnosis of EC absence could be provided only by diagnostic hysteroscopy. However, the absence of symptoms (eg, abnormal uterine bleeding) made it very complicated and ethically inappropriate for such a procedure to be performed.

Although the prevalence in our study was approximately 16-fold greater than the age-matched general population (1.12% vs 0.07%) (eAppendix in the Supplement), a further limitation of our study could be the small number of patients with EC. In fact, only 18 women had an EC-EML score greater than both cutoff values (Youden and 0), with 2 of those patients having false-positive results. In future studies, a very large population (with tens of thousands of participants) or cohorts with participant selection based on EC risk factors or early signs might allow for enrollment of a larger number of patients with EC, improving the diagnostic performance assessment of this screening test. Lastly, owing to the small number of these conditions, our test did not account for the potential confounding role of noncompensated diabetes, ethnicity, and/or concomitant disease. Therefore, additional efforts should be made to enrich the assessed cohort with participants with different conditions, ethnicities, and disease in future studies.

## Conclusions

This study clinically validated a metabolomic-based screening test based on the analysis of DBS. This inexpensive and noninvasive screening system showed good performance (sensitivity, specificity, and accuracy all >99%) in EC detection in postmenopausal women not receiving hormonal therapy. Further studies are needed to enrich the evaluation with participants with different ethnicities, socioeconomical conditions, and comorbidities.

## Notes

Supplement. **eAppendix. **Sample Size Calculation **eFigure. **Enrollment Effect on Metabolomic Signature **eTable 1. **Formulae Used to Evaluate the Performance Parameters **eTable 2. **Confusion Matrix of the Prospective Cohort Study **eReferences.**
