# Supplementary material for: Development and Validation of a Serum Metabolomic Signature for Endometrial Cancer Screening in Postmenopausal Women
Source: JAMA Netw Open. 2020 Sep 28;3(9):e2018327. doi: 10.1001/jamanetworkopen.2020.18327 (PMC7522698; doi:10.1001/jamanetworkopen.2020.18327)
Supplement: Supplement. — eAppendix. Sample Size Calculation eFigure. Enrollment Effect on Metabolomic Signature eTable 1. Formulae Used to Evaluate the Performance Parameters eTable 2. Confusion Matrix of the Prospective Cohort Study eReferences. [file jamanetwopen-e2018327-s001.pdf]

Supplementary Online Content

Troisi J, Raffone A, Travaglino A, et al. Development and validation of a serum metabolomic signature for endometrial cancer screening in postmenopausal women. *JAMA Netw Open*. 2020;3(9):e2018327. doi:10.1001/jamanetworkopen.2020.18327

eAppendix. Sample Size Calculation

eFigure. Enrollment Effect on Metabolomic Signature

eTable 1. Formulae Used to Evaluate the Performance Parameters

eTable 2. Confusion Matrix of the Prospective Cohort Study

eReferences

This supplementary material has been provided by the authors to give readers additional information about their work.

© 2020 Troisi J et al. *JAMA Network Open.*

eAppendix. Sample Size Calculation

Training test

Sample size for the first enrollment aimed to select cases and controls for building the classification models and the ensemble machine learning model. The models were evaluated using the GC-MS serum metabolomic profiles of endometrial cancer patients and controls as reported in Troisi et al. [1]. Minimum sample size required for the pilot study was chosen to obtain ≥ 80% statistical power. Sample size was evaluated using the average power of all metabolites corrected for multiple testing using the false discovery rate (FDR = 0.20) by means of the SSPA package implemented in BioConductor [2] based on Ferreira and Zwinderman algorithms [3]. Figure S1 reported the relationship between simple size and statistical power. Based on this evaluation we decided to enroll at least 50 subjects for each class.

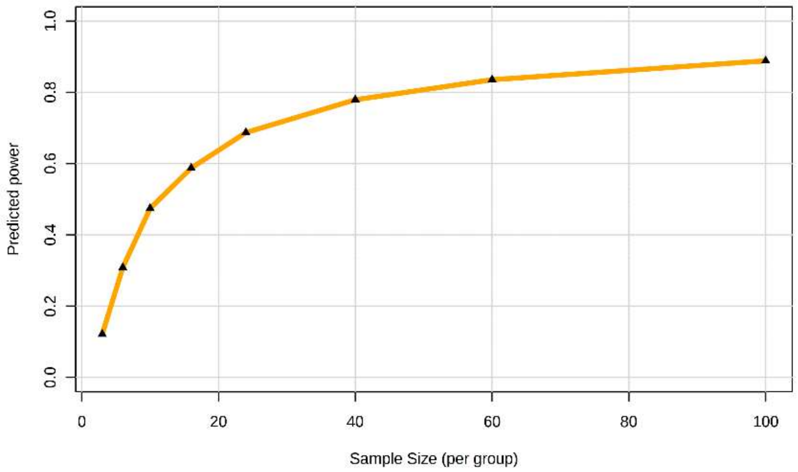

Figure S2: Predicted power and sample size relationship plot

Independent test set

We determined the sample size according to the following equations [4]:

$$N(S) = \frac{1}{p} z^2 \frac{S(1-S)}{w^2} \quad (1)$$

$$N(Sp) = \frac{1}{p} z^2 \frac{Sp(1-Sp)}{w^2} \quad (2)$$

where:

- N(S) is the samples size needed to evaluate the “S” sensitivity of the test
- N(Sp) is the samples size needed to evaluate the “Sp” specificity of the test
- p is the endometrial cancer prevalence among the studied population
- Z is the confidence interval for the number estimation (1.96 for a confidence of 95%)
- w is the alpha value (0.05)

© 2020 Troisi J et al. *JAMA Network Open.*

The endometrial cancer (EC) prevalence in the studied population was estimated based on the Cancer Research UK data [5] and UK population statistics reported by Statista website [6]. Figure S2A and S2B show the EC cases per year and the population size divided for age class, respectively.

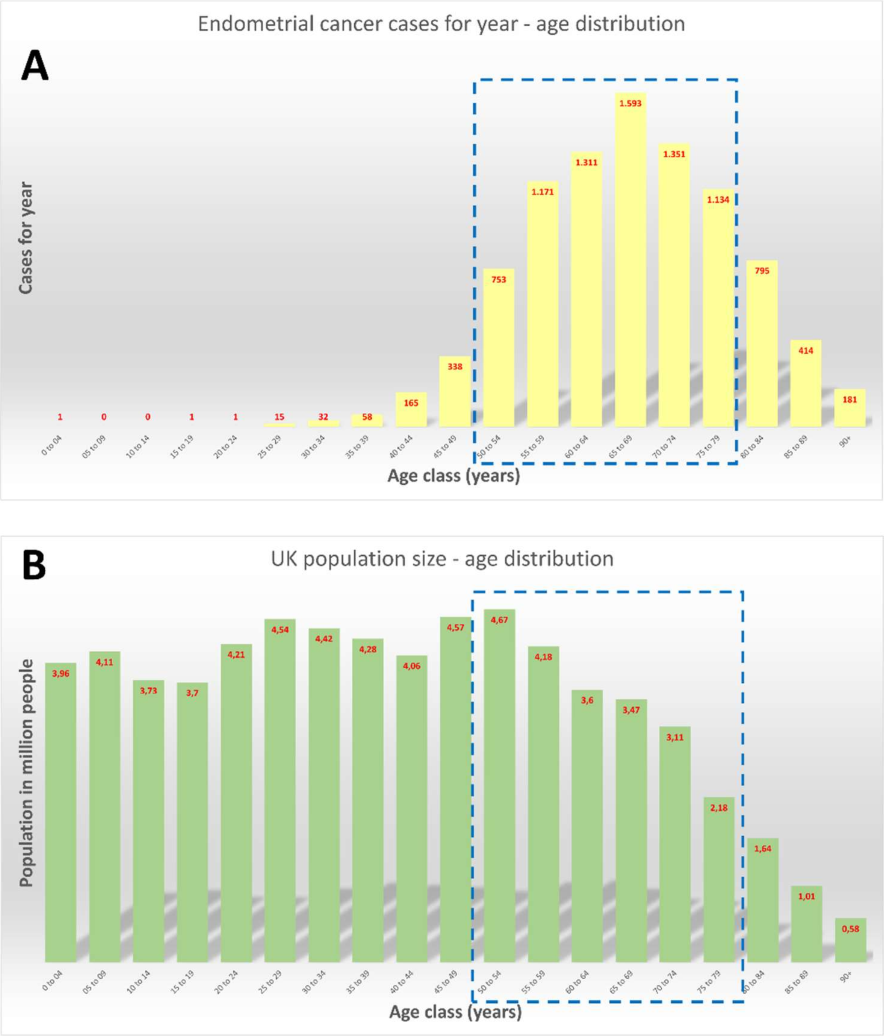

Figure S3: (A) Endometrial cancer new cases per year divided by population age range. The dashed box indicates our study population age range.
(B) United Kingdom population size in million people, divided by population age range. The dashed box indicates our study population age range.

Based on the new cases per year, we have estimated 7313 cases among the women aged between 50-80 years. In the UK there are 21,210,000 persons in this age range. If we assume half are women, the total reference population is 10,605,000 women. Therefore, we estimate in our population of 50-80 year old women, 0.07% have endometrial cancer. Applying equations (1) and (2) we obtained 1043 subjects.

Considering a 30% drop our rate at follow-up we decided to include at least 1350 subjects in the diagnostic performance validation test.

© 2020 Troisi J et al. *JAMA Network Open.*

eFigure. Enrollment Effect on Metabolomic Signature

Principal Component Analysis comparing metabolomes of all enrolled subjects from the two separate enrollments. Data from the EC-affected patients and the matched controls from the first enrollment (Training; n = 120) that were used to train the classification models are shown as purple circles. The green circles represent the subjects of unknown EC-status (Test; n = 1430) that were used to evaluate the performance of the screening test. The high degree of overlap shows that the measured metabolomes of the two separate enrollments are statistically indistinguishable. This illustrates there is no statistical bias resulting from being enrolled in the first or second recruitment.

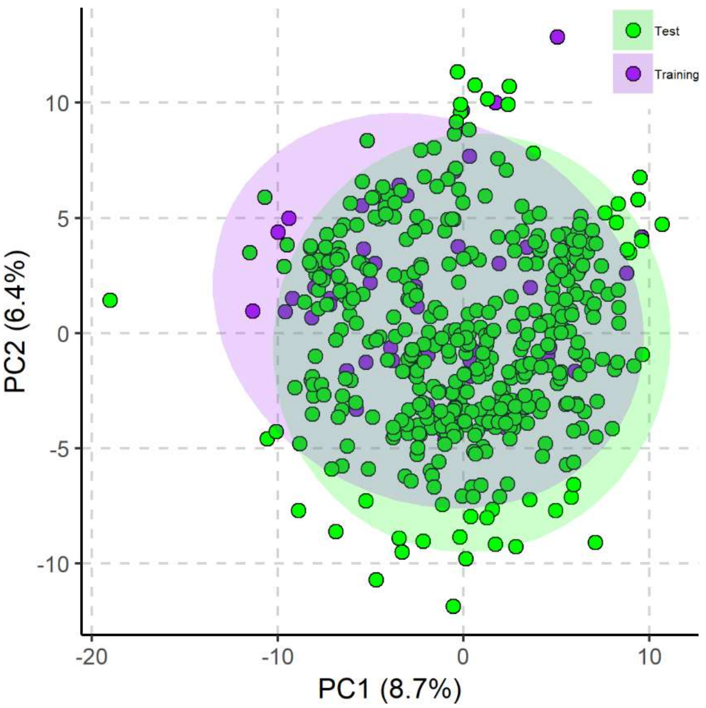

© 2020 Troisi J et al. *JAMA Network Open.*

eTable 1. Formulae Used to Evaluate the Performance Parameters

Mathematical formulas used to calculate various performance metrics of the screening test. Abbreviations: Accuracy (A), False Negative (FN), False Positive (FP), Negative Likelihood Ratio (NLR), Negative Predictive Value (NPV), Positive Likelihood Ratio (PLR), Positive Predictive Value (PPV), Sensitivity (SN), Specificity (SP), True Negative (TN), True Positive (TP).

Parameter	Significance	Value	Standard Error
Sensitivity	The proportion of those who have EC, that were labeled positive by the diagnostic score	$SN = \frac{TP}{TP + FN}$	$\sqrt{\frac{SN(1 - SN)}{(TP + FN)}}$
Specificity	The proportion of those who are disease-free, that were labeled negative by the diagnostic score	$SP = \frac{TN}{TN + FP}$	$\sqrt{\frac{SP(1 - SP)}{(TN + FP)}}$
Positive Predictive Value	The proportion of subjects with a score above the threshold, who truly have EC	$PPV = \frac{TP}{TP + FP}$	$\sqrt{\frac{PPV(1 - PPV)}{(TP + FP)}}$
Negative Predictive Value	The proportion of subjects with score below the threshold who truly do not have EC	$NPV = \frac{TN}{TN + FN}$	$\sqrt{\frac{NPV(1 - NPV)}{(TN + FN)}}$
Positive Likelihood Ratio	Indicates how much the probability of EC increases if the score is above the threshold	$PLR = \frac{SN}{1 - SP}$	
Negative Likelihood Ratio	Indicates how much the probability of EC increases if the score is below the threshold	$NLR = \frac{1 - SN}{SP}$	
Accuracy	The proportion of subjects with a correct diagnosis by the EC score	$A = \frac{TP + TN}{TP + FP + TN + FN}$	

© 2020 Troisi J et al. *JAMA Network Open.*

eTable 2. Confusion Matrix of the Prospective Cohort Study

Confusion matrix of the Youden's selected EC-EML-score cut-off based on prospective cohort study

(A)	True Positive (n)	True Negative (n)
EC-EML-score ≥ cut off value (EC positive subjects)	16	2
EC-EML-score < cut off value (EC negative subjects)	0	1412

© 2020 Troisi J et al. *JAMA Network Open.*

eReferences

1. Troisi, J.; Sarno, L.; Landolfi, A.; Scala, G.; Martinelli, P.; Venturella, R.; Di Cello, A.; Zullo, F.; Guida, M. Metabolomic Signature of Endometrial Cancer. *J Proteome Res* 2018, *17*, 804–812.
2. van Iterson, M.; 't Hoen, P.A.C.; Pedotti, P.; Hooiveld, G.J.E.J.; den Dunnen, J.T.; van Ommen, G.J.B.; Boer, J.M.; Menezes, R.X. Relative power and sample size analysis on gene expression profiling data. *BMC Genomics* 2009, *10*, 439.
3. Ferreira, J.A.; Zwinderman, A. Approximate sample size calculations with microarray data: an illustration. *Stat Appl Genet Mol Biol* 2006, *5*, Article25.
4. Jones, S.R.; Carley, S.; Harrison, M. An introduction to power and sample size estimation. *Emergency medicine journal : EMJ* 2003, *20*, 453–458.
5. Cancer Research UK Uterine cancer incidence statistics 2019.
6. Statista *Mid-year population estimate of the United Kingdom in 2017, by age group (in million people)*; 2019;

© 2020 Troisi J et al. *JAMA Network Open.*
